# The relationship between passion and athlete identity in sport: the mediating and moderating role of dedication

**DOI:** 10.1186/s40359-024-01565-4

**Published:** 2024-02-15

**Authors:** Sinan Uğraş, Barış Mergan, Talip Çelik, Yusuf Hidayat, Cemal Özman, Ümit Doğan Üstün

**Affiliations:** 1 Faculty of Sport Sciences, Çanakkale Onsekiz Mart University, Çanakkale, Turkey; 2 Faculty of Sports Sciences, Tokat Gaziosmanpaşa University, Tokat, Turkey; 3 İnönü University Malatya Vocational School, İnönü University, Malatya, Turkey; 4 Faculty of Sport and Health Education, Universitas Pendidikan Indonesia, Bandung, Indonesia; 5 Faculty of Sports Sciences, Bartın University, Bartın, Turkey; 6 Faculty of Sports Siences, Recep Tayyip Erdoğan Üniversitesi, Rize, Turkey

**Keywords:** Emotion, Commitment in sport, Athlete psychology, Athlete experience, Athlete *character*

## Abstract

**Background:**

In addition to the fact that the concept of passion in sports plays a significant role in the formation of the identity concept of athletes, the dedication of athletes to the sports branches they are interested in also has a significant impact on their passion for the sport they are interested in as well as their identity as an athlete. In this direction, the research aims to investigate the role of dedication as a mediator and moderator in the relationship between athlete identity and passion in sport.

**Methods:**

The research was designed using the quantitative research technique of relational surveying. As data collection instruments for the research, the athlete identity scale, the passion in sport scale, and the sports commitment scale were utilized. 237 amateur and professional athletes, of which 142 were male and 95 were female (Mage = 22.7), participated voluntarily in the study by random sampling. The data were analyzed with the PROCESS and Jamovi programs in order to examine the direct and indirect effects.

**Results:**

Significant effects of sports passion on commitment and athlete identity were found. Since both dedication and athlete identity had a significant effect on passion for sports, it was determined that passion for sports continues to influence athlete identity through the medium of dedication. The moderator significance of medium, high, and low values of devotion was determined.

**Ethics approval number:**

226394, date of registration: 03/11/2022.

**Conclusion:**

On the basis of the results of the statistical analyses, it was determined that the concept of dedication has a mediating and moderating effect on the relationship between sports passion and athlete identity.

## Introduction

It is important to society to observe how players regularly participate in sports without straying from the sports environment in order to develop a habit of sports. In this framework, researchers have extensively investigated the individual identities of athletes. Participation in sports is significantly linked to personal identity [1–2]. An athlete’s relationships and experiences with sports activities can influence their identity. Research indicates that when the concept of identity is psychologically and socially grounded, the greater the emphasis on the role of the athlete, the greater the influence of the individual’s self-esteem, motivation, athletic competence, appearance, success, and performance perceptions on that role [3]. In studies comparing the athlete identity characteristics of those with a strong athlete identity to those with a weak athlete identity, those with a strong athlete identity were found to engage in more physical activity, be more sociable, and have a better health status [4]. When considering the studies on athletes including variables such as retirement [5–7], sports participation level with the intention of permanence in sports [8, 9], sportive performance [10, 11] anxiety and mental resilience [12], it can be said that the concept of athlete identity is a crucial psychological aspect to understand athletes better.

Athlete identity, as examined in several studies, covers self-confidence [13], mental health [14], depression [15], prosocial and antisocial behaviours [16], as well as courage [17] were impacted. In addition to this expression, the fact that the concept of identity functions as a cognitive structure influencing coping strategies, interpreting information, and inspiring harmonious behavior with athlete roles in general demonstrates that the concept of athlete identity is multifaceted [18]. There are also significant research results in the literature that personality traits are associated with critical interpersonal outcomes, including relationship satisfaction, stability, conflict, passion, and dedication [19–21].

This study aimed to investigate the athletes’ passion and dedication to sports as a means to comprehend their continued participation in simple ways. Currently, we consider it advantageous to have an in-depth understanding of the relationship between these two characteristics and athlete identity.

## Theoretical framework

### The relationship between passion and athlete identity

Scientists define passion as an intense affinity, tendency, and motivational concept for an activity that people adore, value, adopt, and invest time and effort in [22, 23]. This definition recalls a famous French maxim: “La sagesse fait durer, les desires font vivre” [24]: " La sagesse fait durer, les desires font vivre”. Indeed, the hearts of human beings beat in rhythm according to the magnitude of their passions [25]. The development of a passion for sports is one of the factors that will aid athletes in overcoming the obstacles and challenges they encounter on their path to success. In this direction, studies on passion in sports have indicated that passion is associated with success in athletes’ performances [22, 26, 27], and it has been revealed that passion is a motivating drive that will provide the energy and determination necessary for athletes to exercise; besides, it can create athletes’ willingness to endure even pain for the sake of sportive performance. On the other hand, the fact that passion in sports encourages athletes to do heavy training and pushes them to compete after numerous pieces of training required to reach the top may indicate that passion in sports is a powerful motivation, and it is stated that passion in sports will also benefit from increasing the athletic performances of athletes [28, 29]. As it represents a powerful motivational force [22, 30], passion may be an essential component in explaining subsequent training behaviours that emerge from complex interactions both during the race and throughout the competition season. It is discussed that some individuals internalize their passion for a particular activity to a significant extent and that passion for the sport they chose may become the centre of their identity [7]. There is also research that passion and identity are interrelated and that passion and identity affect motivational, emotional and behavioural outcomes [22, 23, 31]. Similar to the athlete’s identity, numerous studies indicate that the passion exhibited by athletes stimulates their motivation to participate in sports [32, 33]. The motivational force of passion is expected to have a substantial influence on the identity of athletes. Given the conditions, a hypothesis was developed.

#### H_1_

Passion in sport has a direct positive effect on athlete identity.

### The realitionship between dedication, athlete identity and passion

Dedication in sports can be defined as continuous and consistent experiences in sports environments, including concepts such as belief, effort, energy, and pleasure [34, 35]. It is seen that dedication and dedication are among the factors of athlete personality. Curran et al. [22] stated that dedication is the effort, time and energy spent in moving towards personal goals. As stated by Schaufeli et al. [36], dedication is characterized as a positive state seen together with vigour, internalization and dedication. Wann [37] emphasises that in addition to the physiological and structural factors being crucial for success in sports, another vital factor that is effective in athletes’ continuity in sports is dedication. When the related literature is examined, it is seen that the concept of dedication to the sport, which is associated with dedication, was derived from the Social Exchange Theory proposed by Thibaut and Kelley [38]. According to research, athlete dedication is seen as a permanent, positive, cognitive-emotional experience in sports characterised by confidence, dedication, vigour and enthusiasm [8]. Given this framework, the following hypotheses have been established.

#### H_2_

Dedication has a direct positive effect on athlete identity.

#### H_3_

Passion in sport has a direct positive effect on dedication.

### The current study

Through reviewing the literature, the concept of athlete identity has been examined by the researchers on the following topics: Sports type and achievement [9], athlete personalities of individual and team athletes [39–41], comparison of personality traits of athletes and non-athletes [42, 43], the effect of athletes’ personalities on the relationship between coaches and athletes [44], athlete personalities and mental processes, athletic performance and athlete personality relationship [45], athlete personality and psychology [46]. On the other hand, it is also seen that there are studies examining the passion [47], vitality and performance in sports. On the other part, researchers have reported that there is a negative relationship between their level of dedication, namely their dedication to the sport they are interested in, and burnout [34, 48–50].

In achievement settings, passions and achievement goals are believed to be two important concepts that explain motivated behaviours [51]. Some activities that people enjoy and participate in regularly will be internalized into a person’s identity to the extent that they are highly valued [52, 53]. Posner and Eiler [54] state that there is a strong relationship between intrinsic motivation and passion. Both passion and intrinsic motivation are expressed as one factor [54]. Hollembeak [27] also points out that passion causes the person to work constantly and reach a higher level of performance and is effective in participating in an activity that the she enjoys. The fact that an athlete whose primary goal is to achieve success acts with a determined passion and dedication in this sport branch, regardless of the sports branch s/he has chosen, comes out as a significant criterion [55]. Passion is considerable for an athlete to have high motivation [56] and is one of the most important psychological factors that enable him/her to achieve the highest success [26]. The development of passion in sports will be influential in assisting athletes in overcoming obstacles on their path to success. In this context, it is clear that the concept of passion in sports appears to be a critical source of success in athletes. According to scientific evidence, athlete identity is associated with concepts such as intention to persist and participate in sport, anxiety, endurance, and motivation. In sports, it is seen that dedication is characterized by concepts such as confidence, vigour, and persistence. Given that the concept of passion in sports can be effective in forming, influencing, and shaping athlete identity, the study aims to investigate the mediator and moderator role of the concept of dedication in these two concepts. Given the conditions, the following hypotheses have been established.

#### H_4_

Dedication has a mediating effect on the effect of passion in sport on athlete identity.

#### H_5_

Dedication has a moderating role in the effect of passion in sport on athlete identity.

## Method

### Design

The relational survey model, one of the quantitative research methodologies, is used in this study [57]. The model seeks to ascertain the existence of a relationship between two or more variables, as well as the nature and strength of that relationship [58].

### Data collection tools

In this section of the study, the data collection instruments employed within the scope of the study are described in depth. Initially, the researchers’ demographic information form was utilized. Then, the athlete identity, passion for sport, and the commitment dimension of the sports dedication scale were utilized.

#### Sports commitment scale

The sports commitment scale adapted into Turkish by Cihan et al. [59] consists of a total of 15 questions and 3 dimensions, as well as a scale form adapted as a single dimension. While the sub-dimensions of the sports commitment scale are stated as “fitness”, “dedication” and “internalization”, its unidimensional structure is stated as “dedication”. Within the scope of the research, the dedication dimension of the sports commitment scale was included in the research and used as a data collection tool. The dedication dimension consisted of a total of 5 questions and the internal consistency coefficient was reported as 0.80 [59]. According to the results of the CFA conducted to test the construct validity, it was found to have acceptable values (χ²=14.2/DF = 5, CFI = 0.976, TLI = 0.952, SRMR = 0.0269, RMSEA = 0.0883) [60]. The Cronbach’s α value of the sports dedication sub-dimension was 0.809 and McDonald’s ω value was 0.823. In terms of construct validity and internal reliability coefficients, it was accepted as reliable for this study.

#### Passion in sport scale

The passion in sport scale, which is based on the passion scale developed by Sigmundsson et al. [61] and adapted to sport by Özdayı et al. [62] and validity and reliability studies were conducted, consists of a total of 8 items. In addition to being a scale designed as a 5-point Likert scale graded as “1 = Strongly disagree, 5 = Strongly agree”, the lowest score that can be obtained from the scale is 8 and the highest score is 45 [62]. Confirmatory factor analysis was conducted to determine its suitability for this study. According to the CFA analysis conducted to test the construct validity, it was determined that the RMSEA value was above acceptable values. Therefore, modifications were made between the 4th and 7th and 3rd and 5th items by looking at the residual covariances. After the modification, it was determined that it had acceptable values (χ²=34.7/DF = 18., CFI = 0.978, TLI = 0.966, SRMR = 0.0319, RMSEA = 0.00625) [60]. The Cronbach’s α value of the passion in sport scale was 0.855 and McDonald’s ω value was 0.872. In terms of construct validity and internal reliability coefficients, it was accepted as reliable for this study.

#### Athlete identity scale

The version of the athlete identity scale developed by Brewer and Cornelius [63] and adapted into Turkish by Öztürk and Koca [64] was used. Öztürk and Koca [64] stated that it was appropriate to use the scale adapted into Turkish as a unidimensional scale as well as multidimensional as a result of data analysis. In this direction, the athlete identity scale adapted into Turkish within the scope of the research was used unidimensional. The internal consistency coefficient of the unidimensional scale form was stated as 0.81, and in addition, the lowest score that can be obtained from the scale consisting of 7 items in total is 7, while the highest score is 49 [64]. According to the results of the CFA conducted to test the construct validity, it was found to have acceptable values (χ²=25.3/DF = 13, CFI = 0.947, TLI = 0.914, SRMR = 0.0416, RMSEA = 0.0633) [60]. The Cronbach’s α value of the athlete identity scale was 0.703 and McDonald’s ω value was 0.707. In terms of construct validity and internal reliability coefficients, it was accepted as reliable for this study.

### Research group

The population of the study consists of students who participated in individual and team sports competitions between universities in 2022. In the sample group, the criteria of actively participating in competitions in university teams and being an amateur or professional athlete in a sports club were sought in 2022. Using the convenience sampling method, athletes who met the specified conditions were selected to the sample group on a voluntary basis. In January and March 2023, the data collection process was carried out. The research group consisted of 237 athletes, 95 (40.1%) female and 142 (59.9%) male. Of the athletes participating in the study, 121 (51.1%) were interested in individual sports and 116 (48.9%) were interested in team sports. 204 (86.1%) of the athletes compete in amateur leagues and 33 (13.9%) in professional leagues (Table 1).

**Table 1:** Demographic information of the participants

Variable	n	%
Gender		
Male	142	59.9
Female	95	40.1
Total	237	100
Sport Type		
Individual Sports	121	51.1
Team Sports	116	48.9
Total	237	100
Which leagues do you compete in?		
Amateur League	204	86.1
Professional league	33	13.9
Total	237	100
How many days a week do you train?		
1–3 days	141	59.5
4 and above	96	40.5
Total	237	100
Age	Mean	Sd
	22.27	4.96
How many years have you been doing sports?	Mean	Sd
	7.45	7.00

### Data analysis

This section of the study provides details on the statistical analysis of the collected data. For statistical processing of the obtained data, IBM SPSS 25.0 package program, SPSS PROCESS, and Jamovi 2.2.5 statistical program were utilized. To determine the statistical procedures to be applied to the obtained data, the kurtosis and skewness coefficients (Table 2) were examined to determine if they satisfy the basic assumptions. The fact that the median and arithmetic mean values are close to or equal and the skewness and kurtosis values are within the limits of 2 indicates that the distribution of the participant data is normal [65]. A Pearson correlation analysis was performed to investigate the relationships between passion, commitment, and athlete identity in sport. Following these steps, regression-based mediation analyses were conducted to test the study’s hypotheses [66]. Model 4 in the SPSS PROCESS 4.1 Macro plug-in (The Guilford Press, Calgary, AB, Canada) was used to assess the mediating role of dedication as a mediator between sport passion and athlete identity [67, 68]. PROCESS determines the upper and lower confidence interval limits as a result of bootstrapping, which is the process of re-testing the significance of direct and indirect effects by increasing the sample size, and this limit should not include zero for the indirect effect to be significant. Indirect paths are significant when the 95% confidence interval excludes zero and not significant when the 95% confidence interval includes zero [66]. Accordingly, in order to check the significance of the mediation effect, 5000 bootstrapping (resampling) procedures were applied and the 95% confidence intervals obtained were examined to examine the significance of the indirect effects arising from the mediation analysis. According to the values taken as reference in this study, it was seen that the 95% confidence interval did not include zero. Jamovi 2.2.5 program was preferred to perform statistical procedures in order to test the moderator role of dedication in the effect of passion on athlete identity.

## Results

In this section, the numerical data obtained after the statistical procedures applied to the data obtained from the participants will be presented in tables and interpreted.

### Findings on the mediating effect of dedication on the effect of passion in sport on athlete identity

**Table 2:** Pearson product moment correlation coefficients for total scale scores and normality distribution, mean and standard deviation values of the data

Variables	1	2	$\bar x$	Sd	Skewness	Kurtosis
1- Dedication			4.29	0.60	-0.80	0.22
2- Passion in sport	0.632**		4.15	0.63	-0.69	0.34
3- Athlete identity	0.455**	0.476**	4.96	1.20	-0.19	-0.82

x̄=Mean

Sd = Standard deviation

When Table 2 is examined, it was seen that there was a significant positive relationship between dedication and passion in sport (*r* = .632; *p* < .01) at a medium level and a low level positive relationship with athlete identity (*r* = .455; *p* < .01). In addition, it was determined that there was a low level positive significant relationship between passion in sport and athlete identity (*r* = .476; *p* < .01). When the total mean scores obtained from the scales were examined, it was seen that “Athlete identity” (4.96 ± 1.20), “Passion in sports” (4.15 ± 0.63) and “Dedication” (4.29 ± 0.60).

**Table 3:** Measurament model

				95% Confidance Intervals				
Latent	Observed	Estimate	SE	Lower	Upper	β	z	*p*
Dedication	D1	1.000	0.0000	1.000	1.000	0.645		
	D2	1.341	0.1018	1.142	1.541	0.865	13.18	< 0.001
	D3	1.286	0.1102	1.070	1.502	0.829	11.67	< 0.001
	D4	1.262	0.0957	1.074	1.449	0.814	13.19	< 0.001
	D5	1.201	0.0998	1.005	1.397	0.774	12.03	< 0.001
Athletic Identity	AI1	1.000	0.0000	1.000	1.000	0.603		
	AI2	1.059	0.1161	0.831	1.286	0.639	9.12	< 0.001
	AI3	0.597	0.1181	0.365	0.828	0.360	5.05	< 0.001
	AI4	1.119	0.1327	0.859	1.379	0.675	8.43	< 0.001
	AI5	1.127	0.1216	0.888	1.365	0.680	9.26	< 0.001
	AI6	0.898	0.1295	0.644	1.152	0.542	6.93	< 0.001
	AI7	0.984	0.1472	0.696	1.273	0.594	6.68	< 0.001
Passion in Sport	PS1	1.000	0.0000	1.000	1.000	0.810		
	PS2	1.039	0.0465	0.948	1.130	0.842	22.36	< 0.001
	PS3	0.576	0.0672	0.445	0.708	0.467	8.57	< 0.001
	PS4	1.041	0.0416	0.959	1.123	0.843	24.99	< 0.001
	PS5	0.914	0.0481	1.000	1.009	7410	19.00	< 0.001
	PS6	1.007	0.0481	1.142	1.101	0.816	20.96	< 0.001
	PS7	0.963	0.0500	1.070	1.061	0.780	19.24	< 0.001
	PS8	0.902	0.0557	1.074	1.011	0.730	16.19	< 0.001

Table 3 shows the factor loadings and 95% confidence intervals of the three-factor measurement model used in the research. The CFA results for the construct validity of the three-factor measurement model were found to have acceptable values (χ²=198./DF = 167, CFI = 0.998, TLI = 0.997, SRMR = 0.056, RMSEA = 0.028, NFI = 0.995).

**Table 4:** Main effects on the dependent variable

Variables	Model 1 Dedication			Model 2 Athlete identity			Model 3 Athlete identity		
	B	SE	*p*	B	SE	*p*	B	SE	*p*
(Constant) Passion in sport Dedication	1,800 0.6025	0.2025 0.482	0.000 0.000	1.2311 0.9006	0.4560 0.1086	0.000 0.000	0.3152 0.5936 0.5095	0.514 0.136 0.143	0.000 0.000 0.000
F p R^2^		154.04 < 0.001 0.63			68.77 < 0.001 0.226			42.40 < 0.001 0.2660	

B = Unstandardized beta coefficient

SE = Standard Error

The effects of the independent variables on the dependent variables are given in Table 4. In this table, 3 different sub-models were created in accordance with the model. In Model 1, the effects of passion in sport on dedication were analyzed. Accordingly, it was determined that the effect of passion in sport on dedication was positive (B = 0.6025, *p* < .001), and similarly in Model 2, the effect of passion in sport on athlete identity was positive (B = 0.9006, *p* < .001). In addition, it was determined that dedication had a positive effect on athlete identity (B = 0.5095, *p* < .001).

**Table 5:** Direct and indirect effects of passion in sport on athlete identity

Direct and Indirect Impact			Unstand.	SE		LLCI	ULCI
The total effect of passion in sport on athlete identity			0.9006	0.4560		0.6866	1.1145
The direct impact of passion in sport on athlete identity			0.5936	0.1367		0.3242	0.8630
The mediating effect of dedication on the effect of passion in sport on athlete identity							
Independent	Mediator	Dependent	Unstand.		SE	LLCI	ULCI
Passion in sport >	Dedication >	Athlete identity	0.3070		0.0751	0.1664	0.4626

*The indirect effect is significant if there is no 0 (Zero) between the LLCI and ULCI values

SE = Standard Error

In order to reach the results of the mediation analysis in the study, direct regression analyses were first performed between the variables. The effect of passion in sport on the mediating variable of dedication (Model 1) and its effect on athlete identity was significant (Model 2). In addition, the effect of the independent variables of dedication and passion for sport on athlete identity was found to be significant (Model 3). Therefore, significant relationships necessary for the mediator effect to be present were identified. In other words, it has been determined that a significant relationship continues from passion in sport to athlete identity through the mediator variable of dedication. Accordingly, the mediating effect of dedication was statistically significant (γ=-0.3070, SE = 0.0751, 95% CI [0.1664, 0.4626]) (Table 5.).

**Fig. 1:**
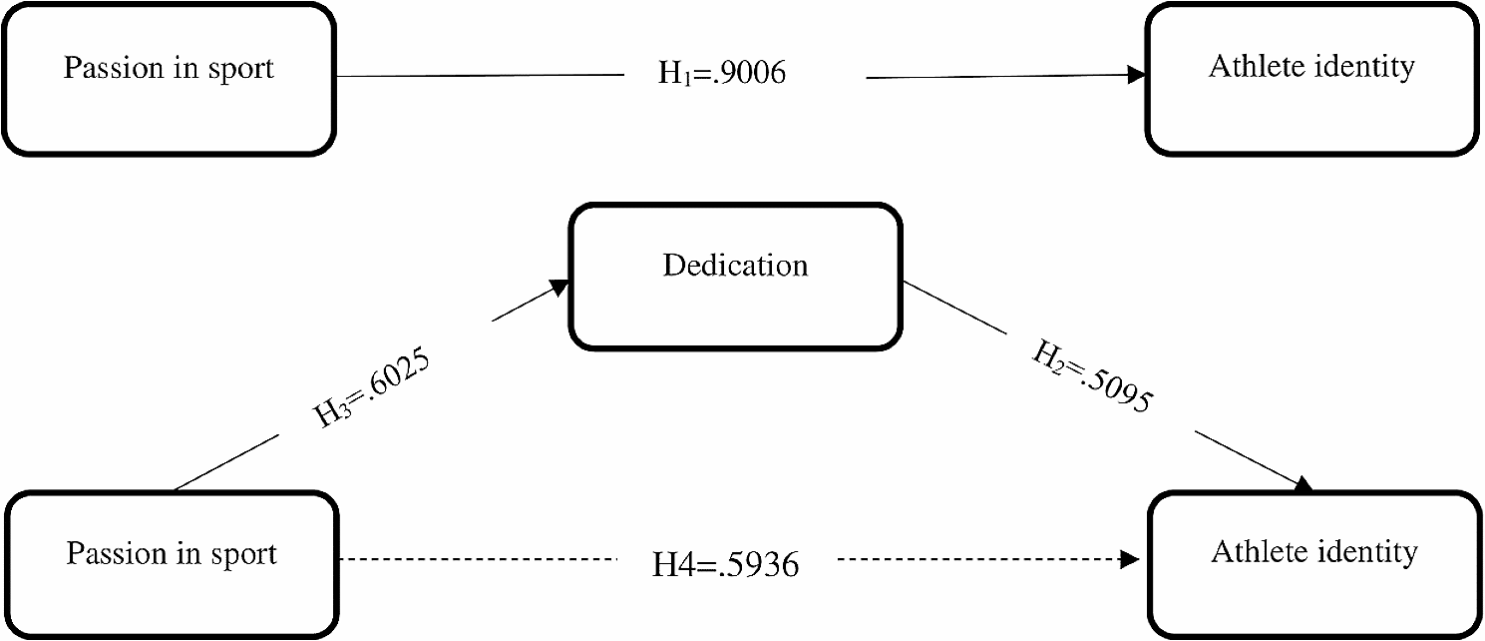
Results of the proposed research model

In Fig. 1, there is a research model showing the mediating role of dedication in the relationship between passion in sports and athlete identity within the scope of the research.

### Findings on the moderating role of dedication in the effect of passion on athlete identity in sport

**Table 6:** The moderator role of dedication in the relationship between passion in sport and athlete identity

			95% Confidence Interval			
Variables	Estimate	SE	Lower	Upper	Z	*P*
Passion in Sport	0.633	0.122	0.3745	0.871	5.21	0.001
Dedication	0.565	0.129	0.3347	0.832	4.39	0.001
Passion in Sport * Dedication	0.327	0.155	0.0555	0.672	2.11	0.035

SE = Standard Error

When Table 6. is examined, it is determined that passion in sport, which is determined as the independent variable in the model, affects the athlete identity positively (Est = 0.633, SE = 0.122, t = 5.21,*p* = .001) and dedication in sport, which is determined as the moderator variable, affects it positively (Est = 0.565, SE = 0.129, t = 4.39,*p* = .001). The moderating effect of sports dedication on the interaction between sports passion and athlete identity was found to be significant (Est = 0.327, SE = 0.155, t = 2.11, *p* = .035).

**Table 7:** Simple slope analysis showing the effects of the moderator role of dedication at different levels in the relationship between passion in sport and athlete identity

			95% Confidence Interval			
Simple Slope	Estimate	SE	Lower	Upper	Z	*P*
Average	0.633	0.121	0.371	0.867	5.23	0.001
Low (-1SD)	0.435	0.138	0.142	0.679	3.16	0.002
High (+ 1SD)	0.831	0.167	0.497	1.164	4.98	0.001

**Fig. 2:**
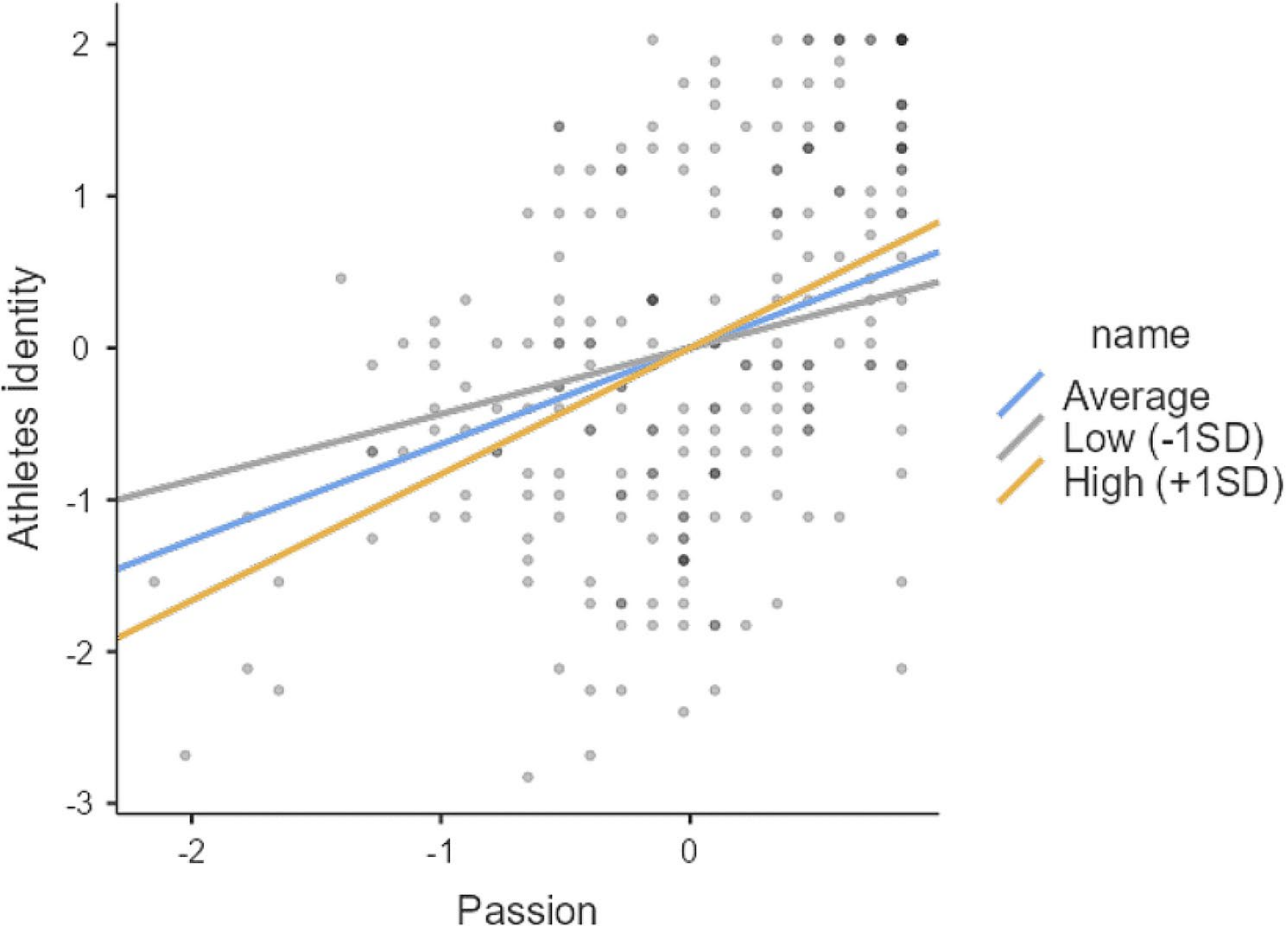
Graphical representation of the moderator effects of dedication

When the details of the moderating effect are examined, it is shown in Table 7 that the moderating effect of sports commitment is significant when it takes medium (Est:0.633, *p* = .001), low (Est:0.435,*p* = .002), high (Est:0.831,*p* = 001) values. According to the results of the slope analysis (simple slope), the effects of the moderator variable are shown in Fig. 2.

## Discussion

The purpose of this study was to investigate the role of the dedication factor as a mediator and moderator of the relationship between passion for sport and athlete identity. Athlete identity as the dependent variable and passion for sport and dedication as independent variables are included in the research. Following statistical analysis, it was determined that the independent variable, passion for sport, positively predicted the dependent variable, athlete identity, and the mediating variable, dedication, also positively predicted the dependent variable, athlete identity. The independent variable, athlete identity, was found to positively predict the dependent variable, passion for sport, and the mediating variable, dedication. On the other hand, it was determined that the effect of sports dedication in the moderator role was substantial. In this section of the study, the statistically derived numerical data were compared with those from the scholarly literature, and inferences were drawn based on their similarities and differences.

The hypothesis that passion in sport has a direct positive effect on athlete identity was confirmed (H_1_). In his research on the concept of passion in sport, aggression, and athlete identity, Donahue et al. [69], found that excessive passion for athletes’ professions can lead to negative outcomes; therefore, when athlete identity is equated with good performance, it can prevent undesirable negative behaviors in sport. When an athlete’s level of passion is directed positively, it can have a positive effect on the athlete’s identity. This positive interaction can result in positive outcomes such as improved performance, motivation, and goal management. According to Gustafsson et al. [56] individuals with harmonious passion control the activity, whereas individuals with obsessive passion are controlled by the activity. The indirect relationship between passion and identity can be expressed in this manner. It is believed that a person’s level of enthusiasm for any activity may indirectly influence the identity-related control mechanism over that activity. In addition to these discourses, Vallerand et al. [23] stated that athletes with an obsessive passion participate in sporting activities in all situations and conditions and cannot refrain from doing so, whereas athletes with a harmonious passion determine their own time while participating in sporting activities and do not engage in the activity under unfavorable conditions. Since identity is related to decision-making skills [70, 71], it can be said that passion indirectly has a positive effect on athlete identity. Lafreniere et al. [72] stated that people with high self-esteem develop a harmonious passion, whereas people with low self-esteem develop an obsessive passion. In the light of the existence of studies indicating that self-concept is related to identity and personality [73–78], it can be said that passion and identity are indirectly related and a harmonious passion can positively affect identity. One of the main characteristics for athletes to continue their sporting careers for a long time is passion. It is an important point for them to do their jobs with passion in order to continue their careers for a longer period of time. Piepiora et al. [79] stated in their study that athletes clarified their athlete identities as the duration of their competitions increased. In this direction, a result confirming the research hypothesis emerges.

The hypothesis that dedication has a direct positive effect on athlete personality was confirmed (H_2_). Physical and athletic activity has a personality-altering effect on those who train [80, 81] Personality traits are associated with long-term sporting success, interpersonal relationships, and the mental states of athletes before, during, and after competitions [82]. Given that athlete identity is a mental structuring of athletes based on numerous internal and external factors, it has significant long- and short-term effects on the athlete. In this context, in the formation of athlete identity, dedication to the sport of interest is viewed as a crucial factor at the outset of the processes required for the athlete’s success. In sports, dedication is the willingness to expend effort and time for personally meaningful goals [22]. When viewed from this perspective, it is clear that dedication plays a crucial role in both training and competition participation. Considering the relationship between athlete identity and goal orientation, it is possible to assert that even indirect dedication has a positive effect on athlete identity.

It was determined that the hypothesis that passion in sport has a direct positive effect on dedication was confirmed (H_3_). According to Lopes and Vallerand [83] harmonious and obsessive passion for sports are positively associated with need satisfaction in sport. Need satisfaction in sports is a phenomenon that encompasses a wide range of concepts, including athletes’ intrinsic and extrinsic motivation, autonomy, feelings of competence, desire for good performance, success, and commitment level. When the concept of dedication is viewed in terms of the athletes’ expenditure of energy, effort, and time on their activity, it can be stated that the concept of passion has a positive effect on dedication in terms of the athletes’ ability to achieve their goals. According to Carpentier et al. [84] a harmonious passion for one’s favorite activity is positively compatible with the flow experienced during the favorite activity and during the studies. Kelecek and Göktürk [85] stated that the athletes’ willingness to fulfill the requirements of the branch, the enjoyment derived from participation in the branch, and the mental occupation associated with the sports branch in which they are interested will not cause them to lose interest in sports, but will facilitate new learning about their branch. In other words, they concluded that athlete commitment has a negative impact on depersonalisation. The findings of this study confirm that a passion for sports has a direct relationship with commitment. It is believed that dedication can explain the athletes’ constant mental occupation, passion for learning, and desire to follow the sports branch they are interested in, thereby preventing emotional states such as boredom and insensitivity in the athlete’s desired sports branch. Vallerand et al. [23] reported that athletes with balanced passion levels did not experience negative situations such as guilt and anxiety when passion and activity were inhibited. This result provides important evidence that harmonious passion among athletes can prevent negative outcomes. Indirectly, it can be stated that a harmonious passion can protect athletes from negative situations and prevent a decrease in their level of commitment to their activity and their goals, i.e., it will have a positive effect.

It was determined that the hypothesis that there is a mediating and moderating effect of dedication on the effect of passion in sports on athlete identity was confirmed (H_4_,H_5_). Researchers have examined the relationship between the concept of athlete commitment, which encompasses the concept of dedication, and other concepts such as motivation, basic psychological needs, burnout, motivational climate, and optimal performance mood, which both direct the individual to the sports environment, provide continuity or cessation of sport, and influence sporting performance [22, 48, 86]. These studies may be an important indicator of the fact that numerous factors influence the dedication of athletes. According to Blecharz and Siekańska [87] sport is beneficial for molding the personality of young athletes. The findings of this study demonstrate that concepts such as training and sport play a role in the formation and formation of the identity of athletes. In this direction, the fact that the athlete’s passion for the sports branch he/she is interested in is effective in his/her participation in training and competition and that dedication is expressed as an important factor for commitment to the sports branch of interest is a significant, albeit indirect, factor in the formation of the athlete’s identity. The fact that passion in sports has a positive effect on an athlete’s identity suggests that the concept of commitment can moderate the positive formation of an athlete’s identity. In his research, Yukhymenko-Lescroart [31], concluded that the autonomous integration of activity into one’s identity will result in a harmonious passion. He explains this by saying that it leads to the complete internalization or incorporation of the activity’s values and rules into the self, accompanied by a sense of willpower, resulting in the participant’s willing participation in the activity. In addition to the fact that this result parallels the finding that passion in sport positively affects athlete identity, it can be stated that the desire of the athlete to voluntarily participate in the activity is related to the level of dedication to the activity, and that the concept of dedication indirectly mediates the positive effect of passion in sport on athlete identity. Examining the personality traits of athletes in the context of their athletic performances reveals research findings related to interpersonal relationships, mental states prior to, during, and after competition, and long-term sports success [82]. As stated previously, the concept of passion in sports affects athletes’ participation in training, performance, pre-competition, and post-competition situations, and the concept of passion in sports affects the identity of the athlete, even indirectly. In this context, the fact that dedication is defined as the time, effort, and energy that athletes devote to achieving their goals [34] and that dedication is a concept that affects athletic performance suggests that it indirectly mediates the positive relationship between passion for sport and athlete identity. Indirectly, the results of a different study [52, 53] support the hypothesis formulated within the scope of this study.

## Conclusion

It is known that athlete identity affects the athlete’s coping strategies, behaviours and decision-making processes. Considering its role in resilience against adversities such as injury in individuals with strong athlete identity and separation from the team, athlete identity should be developed or supported. According to the results of the research, it was determined that passion has a significant effect on athlete identity. It can be stated that the athlete’s passion for his/her work is a supportive factor in shaping his/her athlete identity. According to the other results of the research, it was revealed in the research results that dedication has a mediating and moderating effect on athlete identity. The motivation and goals of the athlete are important determinants of his/her sportive success. Dedication has an important position in extending this motivation and goal commitment over a long period. It can be concluded that the dedication level of the athlete is an important moderator in the formation of athlete identity, and in this context, the higher the level of dedication of the athletes, the easier it will be to reach a strong athlete identity. In cases where the level of dedication is insufficient, the opposite can be considered. It can be suggested that young athletes, especially in lower age groups, and coaches should act knowing that passion and dedication are important supporters in the process of creating an athlete identity. It may be recommended to conduct new research on athlete identity with different variables such as goal commitment, professionalism, and the role of the coach in identity formation.

## Limitations

The fact that the participants included in the study were elite and amateur athletes at the undergraduate level should be considered as a limitation of the study. In future studies, it is thought that repeating the study with different age groups, different cultures, athletes who have been injured, athletes who have achieved sportive success in their sporting life or athletes who have not achieved the desired goals can reduce the limitations of this research and bring a different perspective to the literature. It may be suggested to include different variables other than dedication, passion and athlete identity used in the research. Conducting the research with quantitative research method can be seen as another limitation. It can be thought that the application of mixed research method by adding a qualitative approach to the research will give more depth to the study.

## Data Availability

The datasets used and/or analyses during the current study are available from the corresponding author upon reasonable request.
